# Risk of Second Primary Cancers in Multiple Myeloma Survivors in German and Swedish Cancer Registries

**DOI:** 10.1038/srep22084

**Published:** 2016-02-24

**Authors:** Tianhui Chen, Mahdi Fallah, Hermann Brenner, Lina Jansen, Elias K. Mai, Felipe A. Castro, Alexander Katalinic, Katharina Emrich, Bernd Holleczek, Karla Geiss, Andrea Eberle, Kristina Sundquist, Kari Hemminki, GEKID Cancer Survival Working Group, Karla Geiss, Martin Meyer, Andrea Eberle, Sabine Luttmann, Roland Stabenow, Stefan Hentschel, Alice Nennecke, Joachim Kieschke, Eunice Sirri, Bernd Holleczek, Katharina Emrich, Hiltraud Kajüter, Volkmar Mattauch, Alexander Katalinic, Nora Eisemann, Klaus Kraywinkel, Hermann Brenner, Lina Jansen, Felipe Castro

**Affiliations:** 1 Division of Molecular Genetic Epidemiology, German Cancer Research Center (DKFZ), Heidelberg, Germany; 2 Institute of Occupational Diseases Prevention, Zhejiang Academy of Medical Sciences, Hangzhou, China; 3 Division of Clinical Epidemiology and Aging Research, German Cancer Research Center (DKFZ), Heidelberg, Germany; 4 Division of Preventive Oncology, German Cancer Research Center (DKFZ) and National Center for Tumor Diseases (NCT), Heidelberg, Germany; 5 German Cancer Consortium (DKTK), German Cancer Research Center (DKFZ), Heidelberg, Germany; 6 Department of Internal Medicine V, University Clinic of Heidelberg, Heidelberg, Germany; 7 Institute of Cancer Epidemiology, University of Lübeck, Lübeck, Germany; 8 Cancer Registry of Rhineland-Palatinate, Institute for Medical Biostatistics, Epidemiology and Informatics, University Medical Center, Johannes Gutenberg University, Mainz, Germany; 9 Saarland Cancer Registry, Saarbrücken, Germany; 10 Bayern Cancer Registry, Munich, Germany; 11 Cancer Registry of Bremen, Leibniz-Institute for Prevention Research and Epidemiology - BIPS, Bremen, Germany; 12 Center for Primary Health Care Research, Lund University, Malmö, Sweden; 13 Stanford Prevention Research Center, Stanford University School of Medicine, Stanford, California 94305-5705, USA; 14 Cancer Registry of Berlin and the New Federal States, Berlin, Germany; 15 Hamburg Cancer Registry, Hamburg, Germany; 16 Cancer Registry of Lower Saxony, Hannover, Germany; 17 Cancer Registry of North Rhine-Westphalia, Münster, Germany; 18 Robert Koch Institute, Berlin, Germany

## Abstract

We aimed at investigating the distribution and risk of second primary cancers (SPCs) in multiple myeloma (MM) survivors in Germany and Sweden to provide etiological understanding of SPCs and insight into their incidence rates and recording practices. MM patients diagnosed in 1997–2010 at age ≥15 years were selected from the Swedish (nationwide) and 12 German cancer registries. Standardized incidence ratios (SIRs) were used to assess risk of a specific SPC compared to risk of the same first cancer in the corresponding background population. Among 18,735 survivors of first MM in Germany and 7,560 in Sweden, overall 752 and 349 SPCs were recorded, respectively. Significantly elevated SIRs of specific SPCs were observed for acute myeloid leukemia (AML; SIR = 4.9) in Germany and for kidney cancer (2.3), AML (2.3) and nervous system cancer (1.9) in Sweden. Elevated risk for AML was more pronounced in the earlier diagnosis period compared to the later, i.e., 9.7 (4.2–19) for 1997–2003 period *versus* 3.5 (1.5–6.9) for 2004–2010 in Germany; 3.8 (1.4–8.3) for 1997–2003 *versus* 2.2 (0.3–7.8) for 2004–2010 in Sweden. We found elevated risk for AML for overall, early diagnosis periods and longer follow-up times in both populations, suggesting possible side effects of treatment for MM patients.

Overall survival of multiple myeloma (MM) patients has been improved significantly and the improvement in overall survial was very favorable during the last decade among young patients but more recently extends to older patients as well^1^,^2^,^3^,^4^,^5^,^6^,^7^,^8^. Consequently, the number of second primary cancers (SPCs) in MM survivors has been steadily increasing^1^,^3^,^9^, For instance, elevated risks of acute myeloid leukemia (AML) and myelodysplastic syndromes in MM survivors are well recorded over last decades^1^,^3^,^9^,^10^. Although their exact underlying biologic mechanisms have not been well characterized, treatment-related factors may contribute in addition to inherited genetic predisposition and shared non-genetic factors^1^,^3^,^11^,^12^,^13^.

Melphalan in combination with prednisone (MP) was the standard treatment for all MM patients since the 1960s for almost thirty years^14^. However, the outcomes were unsatisfactory with a median survival of one to three years only. The incorporation of high-dose melphalan (HDM) followed by autologous stem cell transplantation, immunomodulatory drugs (IMiDs, such as thalidomide, lenalidomide and pomalidomide) and proteasome inhibitors (PI, e.g., bortezomib and carfilzomib) in MM treatment paved the road towards a sustained disease control and markedly improved survival^1^,^4^,^15^,^16^,^17^,^18^,^19^,^20^. Whereas induction therapy incorporating at least one novel agent (IMiD or PI) followed by HDM remains the standard care for younger MM patients (<70 years), MP in combination with either thalidomide or bortezomib, or lenalidomide in combination with dexamethasone, is the preferred treatment for elderly MM patients^21^,^22^,^23^.

Whether or not treatment of MM patients will increase the risk of other SPCs remains under-investigated and, population-based large-scale studies are highly warranted because previous studies were limited by small numbers of MM patients and of SPCs in MM survivors^1^,^3^,^9^,^10^. However, these studies demonstrated that exposure to alkylating agents such as melphalan/HDM with or without lenalidomide might increase risk of SPC^4^. To our knowledge, investigations on the risk of specific SPC in MM survivors in two different populations have not been reported. Therefore, using the latest version of the pooled database from 12 population-based German cancer registries^24^ and the nationwide Swedish Family-Cancer Database (FCD)^25^, we aimed at investigating the risk of specific SPCs in MM survivors in the two populations, which provides insight into the etiology of SPCs in MM survivors (particularly regarding possible side effects of MM treatment) and into registration practices of SPCs in the two populations.

## Results

### Distribution of specific SPCs in MM survivors in the two populations

Overall numbers of patients diagnosed with first primary MM during 1997–2010 and aged ≥15 years were 18,735 in Germany and 7,560 in Sweden, accounting for 1.2% and 1.3% of all first primary cancers (except non-melanoma skin cancer), respectively. Among these patients, 752 and 349 SPCs were detected in Germany and Sweden, respectively. The distribution of specific SPCs after MM is presented in Table 1. Overall, frequency ranking order of a specific SPC was quite similar in Germany and Sweden, i.e., the ranking order of the two most frequent SPCs was identical in Germany and Sweden in the sequence of prostate and colorectal cancers, while the ranking order of other SPCs was generally similar, except for stomach cancer [5th (5.5%) in Germany versus 13th (2.3%) in Sweden)] and cancers of the nervous system [13^th^ (1.6%) in Germany versus 9^th^ (4.9%) in Sweden)]. We found similar distributions of characteristics of MM patients in Germany compared to Sweden, e.g., for percentage of men (51.9% in Germany *versus* 54.2% in Sweden), mean age at diagnosis [69 years (range: 15–102 years) in Germany *versus* 70 years (range: 24–98 years) in Sweden], and mean follow-up time after first MM until SPCs, death, or end of the study, whichever came first [2.6 years (range: 0–13 years) in both countries].

### Risk of a specific SPC in MM survivors in the two populations

The overall SIR and further stratification by the study period are presented in Table 2. We found SIRs to be elevated in Germany for leukemia (SIR = 1.7; 95% CI, 1.2–2.4; out of 14 cancers in total) only and in Sweden for all SPCs combined [1.3 (1.2–1.4)], kidney cancer [2.3 (1.3–3.7)], nervous system cancer [1.9 (1.1–3.1)] and leukemia [1.6 (1.0–2.4)]; elevated SIR for leukemia in both countries was actually due to AML [4.9 (3.2–7.3) in Germany versus 2.3 (1.2–4.0) in Sweden]. Decreased SIRs at levels ranging from 0.4 to 0.9 were found only in Germany for the combined SPCs and six cancers (colorectal, lung, breast, endometrial, prostate and urinary bladder cancers). Further stratification by study period (Table 2) showed similar SIRs in two study periods in Germany and Sweden, except for the combined SPCs [1.2 (1.1–1.4) in 1997–2003 period *versus* 0.9 (0.8–0.96) in 2004–2010 period) and colorectal cancer [1.3 (0.9–1.8) versus 0.5 (0.4–0.7)] in Germany; although the number of AML cases was small, elevated risk for AML was more pronounced in the earlier diagnosis period compared to the later, i.e., 9.7 (4.2–19) for 1997–2003 *versus* 3.5 (1.5–6.9) for 2004–2010 in Germany; 3.8 (1.4–8.3) for 1997–2003 *versus* 2.2 (0.3–7.8) for 2004–2010 in Sweden. Additionally, SIRs for any SPCs were similar between men and women in both countries (data not shown).

We observed a declining trend of SIRs along with increasing age at diagnosis of first primary MM for most of SPCs in Germany, but not in Sweden (Table 3). Elevated SIRs were found in Germany for diagnosis age <65 years for some cancers such as AML [14 (8.0–24)], stomach cancer [2.4 (1.4–3.8)] and kidney cancer [1.9 (1.1–3.0)] and for diagnosis age at 65–74 years for AML [3.1 (1.1–6.8)] only, while in Sweden elevated SIRs were found for diagnosis age <65 years for kidney cancer [4.2 (1.1–11)] only, for diagnosis age at 65–74 years for the combined SPCs [1.4 (1.1–1.7)] and AML [2.9 (1.2–6.0)], and for diagnosis age ≥75 years for the combined SPCs [1.2 (1.0–1.5)] and nervous system cancer [4.0 (1.5–8.6)]. Nevertheless, decreased SIRs at levels ranging from 0.3 to 0.9 were found only in Germany for some cancers by the stratification of age at diagnosis (Table 3).

The SIRs stratified by follow-up time after first MM (<1 year, 1–4 and ≥5 years) are presented in Table 4. Although there was no specific pattern of SIRs by follow-up time in Germany and Sweden, elevated SIRs were found in Germany for AML in ≥5 years follow-up (8.2) and 1–4 years follow-up (5.0), while in Sweden elevated SIRs were found in ≥5 years follow-up for the combined SPCs (1.3) only, in 1–4 years follow-up for AML (2.7), non-Hodgkin lymphoma (2.2), melanoma (1.9), liver and gallbladder cancer (1.6), and the combined SPCs (1.4), and in <1 year follow-up for kidney cancer (3.1) and nervous system cancer (3.0). Nevertheless, decreased SIRs at levels ranging from 0.2 to 0.9 were found only in Germany for some cancers by the stratification of follow-up time (Table 4).

Additionally, sensitivity analyses restricted to eight German cancer registries with full follow-up period 1997–2010 did not essentially change our results (Appendix Tables 1, 2, 3, [Table t1], [Table t1], [Table t2], [Table t2], [Table t2], [Table t3]). We therefore reported data based on 12 German cancer registries to ensure a larger sample size.

## Discussion

Basic demographic and epidemiological data, such as incidence and survival rates of first primary MM, should be known for the investigation on SPC rates in MM survivors in two populations. Firstly, age-standardized incidence rate of MM (European standard) in 2012 was similar in Germany compared to Sweden for both men and women (5.4 per 100,000 for German men *versus* 5.3 for Swedish men; 3.5 for German women *versus* 3.6 for Swedish women)^26^. Secondly, survival rate after the diagnosis of first MM could be a relevant parameter. Age-standardized 5-year relative survival of MM in the 1995–1999 period was lower in Germany (29%) compared to Sweden (40%)^27^. Thanks to the widespread introduction of novel agents in addition to conventional chemotherapy and high dose melphalan (HDM)^2^,^19^,^20^, it reached 45% for German men in 2010^28^ versus 47% for Swedish men for the 2009–2012 period and 45% for women from both countries, respectively (NORDCAN)^29^,^30^. Thus, MM survival before 2000 was lower in Germany compared to Sweden but the difference has narrowed down thereafter.

A major strength of our study is the design of population-based investigations on risk of a specific SPC in MM survivors in two European populations. Another strength is using large high-quality databases (covering approximately 27 and 9 million people of the German and Swedish population, respectively), including very large samples of MM patients (18,735 from Germany and 7,560 from Sweden). One limitation concerns lack of detailed clinical and/or treatment data, and information on the MM subtypes or prognostic factors such as cytogenetics; however, population-based cancer registries commonly do not have this kind of information. Because sensitivity analyses restricted to eight German cancer registries with full follow-up period 1997–2010 did not essentially change our results (Appendix Tables 1, 2, 3, [Table t1], [Table t1], [Table t2], [Table t2], [Table t2], [Table t3]), we herein presented results according to the data from 12 German cancer registries after taking this difference (four German registries started cancer registration later than 1997) into account for person-year calculations in the German dataset.

Our findings of similar distribution of specific SPCs in the two populations may suggest common etiology for most of SPCs and similarities in the registration for these cancers in the two populations. Our findings of 1.3-fold elevated risk for the combined SPCs in Sweden are consistent with a previous report with partly overlapping data (1986–2005) showing 1.26-fold elevated risk of developing any second malignancies in MM patients, compared to Swedish general population^10^. Our finding of elevated risk for AML in both countries (4.9 in Germany versus 2.3 in Sweden) is consistent with previous reports^1^,^3^,^9^,^10^, while elevated risk for AML was also found on longer follow-up and earlier diagnosis periods (Table 4). Exposure to alkylating agents (e.g., melphalan and cyclophosphamide) with or without IMiDs as previously reported might sensibly contribute to both observations^1^,^4^. The use of alkylating agents was very common in the earlier diagnosis periods because no other therapeutic options existed. Therefore, these patients might have been exposed to high doses of alkylating agents in the earlier years of diagnosis. However, after the dawn of the new millennium, alkylating agents might not have been used as intensively and frequently as in earlier diagnosis periods because of the introduction of the novel agents. In the later periods, survival from MM diagnosis improved markedly and long-term survivors from both diagnosis periods accumulated. Therefore, AML as long-term side effect from alkylating agents, as described from other cancer entities such as testicular and ovarian cancers^31^, was more likely to evolve on long-term follow-up. This is supported by the fact that the World Health Organization (WHO) defines subtypes of AML as a second primary malignancy associated with the exposure to alkylating agents^32^. Nevertheless, non-treatment factors such as inherited genetic predisposition, multiple myeloma-related factors and enviromental factors might also contribute, likely in combination with treatment factors^3^. Further investigations on AML with larger sample size in MM survivors shall be warranted.

Our findings of elevated SPC risk for kidney and nervous system cancers are principally consistent with previous reports of elevated risk in a single country^1^,^3^. Elevated risks for kidney and nervous system cancers were most likely due to increased medical surveillance after the diagnosis of first MM, as suggested by our finding of the elevation found only in <1 year follow up (Table 4). The etiology of SPCs in MM survivors is most likely a multifactorial process and has not been well understood^3^. While the disease of MM itself is associated with elevated risk of second cancers^4^,^10^, other risk factors could also contribute, e.g., characteristics of MM patients, shared lifestyle risk factors between first MM and a SPC, and genetic factors involved in an individual’s susceptibility to development of second primary malignancies, as suggested by other studies^3^,^4^,^9^,^30^,^33^.

Decreased SIRs at levels ranging from 0.4 to 0.9 were observed only in Germany for some cancers, including combined SPCs, prostate and colorectal cancers (Table 2), which might be attributed to reporting practices in Germany; prostate and colorectal cancers were two most frequent SPCs in both populations (Table 1). Nevertheless, observed SIRs below 1.0 could result from insufficient medical care rather than incomplete reporting because in the presence of MM with poor prognosis, especially before 2000 in Germany^2^,^27^ , no extended diagnostic efforts for SPCs were made in MM patients in Germany. Furthermore, missed deaths data could also contribute to SIR<1.0.

In conclusion, our study provides a comprehensive overview on the risk of a specific SPC in MM survivors, using high-quality data from German and Swedish population-based cancer registries. Although not all SIRs in subgroup-specific analyses reached statistical significance, elevated risks for AML were found for overall, early period and longer follow-up time in both populations, suggesting side effects of treatment for MM patients. Since elevated risks for kidney and nervous system cancers were most likely due to increased medical surveillance after the diagnosis of first MM, there was no consistent evidence on cancer other than AML in MM survivors.

## Methods

### German data

Details on the pooled German database were described elsewhere^24^. Briefly, data were originally collected from population-based cancer registries covering 13 of 16 German federal states. According to some criteria closely related to data quality, such as the proportions of cancer cases notified by death certificate only (DCO) or autopsy only (those patients were excluded from the analyses), data from 12 cancer registries, covering a population of 26.7 million people (33% of the total German population), were retained in the pooled German database for further analyses^24^. According to the rules set up by the International Agency for Research on Cancer (IARC) (16), German cancer registries commonly did not register tumors occurring at the same organ or at the contralateral organ for SPCs and, non-melanoma skin cancers were not continuously collected. For comparability and consistency, second non-melanoma skin cancer and second MM were therefore not included in this study. Cancers were recorded according to the International Classification of Diseases, 10^th^ version (ICD-10)^34^ and the percentage of microscopically verified cancer diagnosis was larger than 95% in all registries^24^. Patients who were diagnosed with MM as first primary cancer between 1997–2010, with an age of ≥15 years and with a follow-up information until the end of December 2010 were included in the current analyses.

### Swedish data

Swedish FCD was used for the current study; details on this database were described elsewhere^25^. For comparability and consistency, the same criteria used for German data were adopted for Swedish data, e.g. the definition of primary cancers was recoded and restricted to the study period 1997–2010. Briefly, we used all Swedish MM patients diagnosed 1997–2010, covering approximately 9 million Swedes. DCO cases, second non-melanoma skin cancer and second MM were excluded and, age at diagnosis of first MM and SPC cases was restricted to ≥15 years. Information on cancer cases was retrieved from the Swedish Cancer Registry for the years 1997–2010, relying on separate compulsory notifications from clinicians, pathologists and cytologists^35^; cancers during the study period were recorded according to both ICD-7 and ICD-10 codes. The Swedish Cancer Registry only records primary malignancies. Metastasized cancers to other sites were only registered at primary sites and, for multiple primary cancers occurring in same organ or same organ system, only clearly separated malignancies were accepted as multiple primaries and registered^36^. Close to 100% of the registered neoplasms were histologically verified and approximately 98% of second neoplasms were correctly verified according to a re-evaluation study of 209 multiple primary tumors^35^.

### Statistical analyses

For both German and Swedish datasets, standardized incidence ratios (SIRs), calculated as the ratio of observed to expected numbers of cases, were used to assess the risk of a specific SPC in MM survivors. The expected number of a SPC in MM survivors was calculated from the strata-specific first same cancer incidence rates in the Swedish and German general population, respectively, multiplied by the corresponding person-years in MM survivors. Person-years at risk were accumulated for each patient, starting at the date of diagnosis of the first MM (diagnosed from 1997 to 2010), and terminating on the date of a SPC, date of death, date of emigration, or December 31^st^, 2010 (end of the study), whichever came earliest.

All SIRs for Germany and Sweden were adjusted for three identical variables [sex, age (5-year bands), and calendar period (1995–2000, 2001–2005, and 2006–2010)] and a regional category (12 states in Germany and 4 categories in Sweden). The 95% confidence intervals (CIs) for SIRs were calculated assuming that the cases followed a Poisson distribution. Statistical significance for SIRs to be higher or lower than 1.00 was derived from whether or not the 95% CIs for those SIRs included 1.00. We further stratified patients by years of diagnosis (1997–2003 and 2004–2010), and considered their “restricted” follow-up time in 1997–2003 and 2004–2010, respectively. Additional stratifications by characteristics of cancer patients [sex, age at diagnosis of MM (<65, 65–74 and ≥75 years), and follow-up time after MM (<1 year, 1–4 and ≥5 years)] were also conducted. In order to avoid chance findings, we set up rules for showing results: Firstly, only cancer sites with a total number of a specific SPC ≥5 cases in both countries (except subtypes of leukemia) are presented in the tables. Secondly, although we generally used 1-digital number for reporting SIRs and 95% CIs, for those 95% CIs very close to 1.0, the digital number depends on whether it could be distinguished from 1.0. For instance, 0.8–0.96 will replace 0.8–1.0 in the manuscript text. Additionally, sensitivity analyses restricted to eight German cancer registries with full follow-up period 1997–2010 were conducted because four German registries started cancer registration later than 1997. SAS software (version 9.3, SAS Institute Inc., Cary, NC) was used for the data analyses. Data collection within the German Population-Based Cancer Registries was carried out according to state cancer registry laws and, within this project, only completely anonymous data transferred from the cancer registries were analyzed; although the data in the Swedish Family-Cancer Database were completely anonymous and their use did not entail ethical problems either, ethical approval by the Institutional Review Board, Karolinska Institute was obtained.

## Additional Information

**How to cite this article**: Chen, T. *et al*. Risk of second primary cancers in multiple myeloma survivors in German and Swedish cancer registries. *Sci. Rep*. **6**, 22084; doi: 10.1038/srep22084 (2016).

## Figures and Tables

**Table 1 t1:** Number of second primary cancers in multiple myeloma survivors in Germany (n = 18,735) and Sweden (n = 7,560).

Sites of second cancers	Germany		Sweden		%		Rank$,#	
	N	Per 1000+	N	Per 1000+	Germany	Sweden	Germany	Sweden
Prostate	117	6.2	65	8.6	15.6	18.6	1	1
Colorectum	104	5.6	57	7.5	13.8	16.3	2	2
Lung	84	4.5	22	2.9	11.2	6.3	3	5
Breast	69	3.7	30	4.0	9.2	8.6	4	3
Stomach	41	2.2	8	1.1	5.5	2.3	5	12
Leukemia	35	1.9	24	3.2	4.7	6.9	6	4
Kidney	35	1.9	17	2.2	4.7	4.9	7	8
Melanoma	31	1.7	22	2.9	4.1	6.3	8	6
Urinary bladder	23	1.2	19	2.5	3.1	5.4	9	7
Liver and gallbladder	23	1.2	6	0.8	3.1	1.7	10	13
Non-Hodgkin lymphoma	22	1.2	16	2.1	2.9	4.6	11	10
Unknown primary	16	0.9	10	1.3	2.1	2.9	12	11
Nervous system	12	0.6	17	2.2	1.6	4.9	13	9
Endometrium	8	0.4	5	0.7	1.1	1.4	14	14
Total combined^a^	752	40.1	349	46.2	100.0	100.0		

Only cancers with at least 5 SPC cases are presented.

^a^Including cancers not presented individually.

^+^Overall per 1000 population afflicted with each type of cancer.

^$^Frequency rank.

^#^There was no significant difference (p-value = 0.84) in rank between Germany and Sweden tested by Wilcoxon two-sample test using PROC NPAR1WAY procedure.

**Table 2 t2:** SIRs of second primary cancers in survivors of multiple myeloma (MM) in Germany and Sweden for overall and by study period*.

Sites of second cancers	Germany									Sweden								
	1997–2003			2004–2010			Overall			1997–2003			2004–2010			Overall		
	N	SIR	95%CI	N	SIR	95%CI	N	SIR	95%CI	N	SIR	95%CI	N	SIR	95%CI	N	SIR	95%CI
Stomach	9	1.2	(0.5–2.2)	21	1.3	(0.8–2.0)	41	1.1	(0.8–1.5)	5	1.3	(0.4–3.1)	2	0.9	(0.1–3.3)	8	1.4	(0.6–2.7)
Colorectum	35	1.3	(0.9–1.8)	33	0.5	(0.4–0.7)	104	0.7	(0.6–0.9)	20	1.0	(0.6–1.5)	22	1.1	(0.7–1.7)	57	1.3	(1.0–1.7)
Liver and gallbladder	4	0.9	(0.2–2.3)	15	1.4	(0.8–2.3)	23	1.0	(0.6–1.4)	3	1.4	(0.3–4.0)	1	2.8	(0.1–16)	6	1.5	(0.5–3.2)
Lung	18	0.9	(0.5–1.4)	41	0.9	(0.6–1.2)	84	0.8	(0.6–1.0)	9	1.0	(0.5–1.9)	6	1.6	(0.6–3.6)	22	1.3	(0.8–2.0)
Breast	13	0.6	(0.3–1.1)	34	0.7	(0.5–1.0)	69	0.7	(0.6–0.9)	11	0.9	(0.5–1.7)	9	1.0	(0.5–1.9)	30	1.1	(0.8–1.6)
Endometrium	4	1.0	(0.3–2.6)	3	0.4	(0.1–1.0)	8	0.4	(0.2–0.8)	2	1.0	(0.1–3.5)	0	0.0		5	1.0	(0.3–2.4)
Prostate	27	1.1	(0.7–1.6)	62	0.8	(0.6–1.0)	117	0.7	(0.6–0.9)	16	0.7	(0.4–1.2)	27	1.6	(1.1–2.4)	65	1.2	(0.9–1.6)
Kidney	10	1.6	(0.8–2.9)	16	1.1	(0.6–1.8)	35	1.1	(0.7–1.5)	8	2.9	(1.3–5.8)	7	1.8	(0.7–3.8)	17	2.3	(1.3–3.7)
Urinary bladder	6	0.7	(0.3–1.5)	9	0.5	(0.2–0.9)	23	0.5	(0.3–0.8)	3	0.7	(0.1–2.0)	10	1.5	(0.7–2.8)	19	1.0	(0.6–1.5)
Melanoma	8	2.2	(1.0–4.4)	12	0.7	(0.3–1.2)	31	1.3	(0.9–1.9)	6	1.3	(0.5–2.8)	9	1.3	(0.6–2.5)	22	1.4	(0.9–2.1)
Nervous system	4	2.3	(0.6–5.9)	5	1.2	(0.4–2.7)	12	1.3	(0.6–2.2)	9	2.0	(0.9–3.9)	5	1.2	(0.4–2.7)	17	1.9	(1.1–3.1)
Non–Hodgkin lymphoma	5	1.1	(0.3–2.5)	11	0.9	(0.4–1.6)	22	0.8	(0.5–1.3)	6	1.4	(0.5–3.1)	4	1.7	(0.5–4.3)	16	1.5	(0.9–2.5)
Leukemia	12	3.2	(1.6–5.6)	12	1.3	(0.7–2.3)	35	1.7	(1.2–2.4)	8	1.7	(0.7–3.4)	9	1.9	(0.9–3.5)	24	1.6	(1.0–2.4)
Myeloid leukemia	11	7.7	(3.8–14)	10	2.6	(1.3–4.9)	31	3.8	(2.6–5.4)	6	3.0	(1.1–6.5)	3	1.7	(0.3–4.8)	14	2.0	(1.1–3.3)
*Acute myeloid leukemia*	8	9.7	(4.2–19)	8	3.5	(1.5–6.9)	24	4.9	(3.2–7.3)	6	3.8	(1.4–8.3)	2	2.2	(0.3–7.8)	13	2.3	(1.2–4.0)
*Chronic myeloid leukemia*	1	2.3	(0.1–13)	0			1	0.5	(0.0–2.6)	0	0.0		0	0.0		0	0.0	
Unknown primary	2	0.6	(0.1–2.1)	11	1.4	(0.7–2.4)	16	0.9	(0.5–1.4)	6	1.0	(0.4–2.3)	2	1.6	(0.2–5.9)	10	1.5	(0.7–2.8)
Total combined^a^	193	1.2	(1.1–1.4)	350	0.9	(0.8–1.0)	752	0.9	(0.8–0.9)	124	1.0	(0.9–1.2)	124	1.3	(1.1–1.6)	349	1.3	(1.2–1.4)

Only cancers with at least 5 SPC cases (except interested subtypes of leukemia) are presented.

^*^Rounding off for SIRs above 10; Bold type (elevated risk) and underscored type (decreased risk): 95% CIs did not include 1.00.

^a^Including cancers not presented individually.

**Table 3 t3:** SIRs of second primary cancers in survivors of multiple myeloma (MM) by age at diagnosis of first MM in Germany and Sweden*.

Sites of second cancers	Germany									Sweden								
	<65 yrs			65–74 yrs			≥75 yrs			<65 yrs			65–74 yrs			≥75 yrs		
	N	SIR	95%CI	N	SIR	95%CI	N	SIR	95%CI	N	SIR	95%CI	N	SIR	95%CI	N	SIR	95%CI
Stomach	18	2.4	(1.4–3.8)	11	0.8	(0.4–1.4)	12	0.8	(0.4–1.4)	0	0.0		2	2.3	(0.3–8.4)	6	1.3	(0.5–2.8)
Colorectum	24	0.8	(0.5–1.2)	41	0.7	(0.5–1.0)	39	0.7	(0.5–1.0)	8	1.0	(0.4–1.9)	19	1.5	(0.9–2.3)	30	1.3	(0.9–1.9)
Liver and gallbladder	2	0.4	(0.0–1.4)	13	1.3	(0.7–2.3)	8	0.9	(0.4–1.8)	0	0.0		1	1.3	(0.0–7.2)	5	1.6	(0.5–3.7)
Lung	23	0.8	(0.5–1.2)	36	0.8	(0.6–1.1)	25	0.9	(0.6–1.3)	10	1.4	(0.7–2.6)	7	1.3	(0.5–2.7)	5	1.1	(0.3–2.5)
Breast	21	0.7	(0.4–1.1)	28	0.8	(0.5–1.2)	20	0.7	(0.4–1.1)	12	0.8	(0.4–1.5)	10	1.7	(0.8–3.0)	8	1.3	(0.6–2.5)
Endometrium	4	0.8	(0.2–2.0)	2	0.3	(0.0–0.9)	2	0.3	(0.0–1.1)	4	1.5	(0.4–3.9)	0	0.0		1	0.6	(0.0–3.4)
Prostate	26	0.6	(0.4–0.9)	63	0.8	(0.6–1.1)	28	0.7	(0.4–1.0)	17	1.1	(0.6–1.7)	21	1.4	(0.9–2.2)	27	1.2	(0.8–1.7)
Kidney	18	1.9	(1.1–3.0)	13	1.0	(0.5–1.6)	4	0.4	(0.1–1.1)	4	4.2	(1.1–11)	6	2.1	(0.8–4.5)	7	2.0	(0.8–4.1)
Urinary bladder	5	0.7	(0.2–1.5)	8	0.5	(0.2–0.9)	10	0.6	(0.3–1.1)	2	1.3	(0.2–4.5)	9	1.4	(0.6–2.6)	8	0.7	(0.3–1.4)
Melanoma	11	1.5	(0.8–2.7)	13	1.4	(0.8–2.4)	7	1.0	(0.4–2.1)	2	0.8	(0.1–3.0)	18	1.5	(0.9–2.4)	2	1.3	(0.2–4.7)
Nervous system	6	1.9	(0.7–4.1)	5	1.3	(0.4–3.0)	1	0.4	(0.0–2.4)	5	2.0	(0.6–4.6)	6	1.3	(0.5–2.8)	6	4.0	(1.5–8.6)
Non–Hodgkin lymphoma	5	0.8	(0.3–1.8)	11	1.1	(0.5–1.9)	6	0.7	(0.2–1.4)	3	1.1	(0.2–3.3)	4	1.3	(0.4–3.4)	9	1.9	(0.9–3.6)
Leukemia	20	4.2	(2.5–6.4)	11	1.4	(0.7–2.4)	4	0.6	(0.2–1.4)	6	1.7	(0.6–3.8)	9	2.0	(0.9–3.8)	9	1.3	(0.6–2.5)
Myeloid leukemia	19	11	(6.3–16)	8	2.5	(1.1–4.9)	4	1.3	(0.4–3.4)	3	1.3	(0.3–3.9)	7	2.5	(1.0–5.2)	4	1.9	(0.5–4.8)
*Acute myeloid leukemia*	15	14	(8.0–24)	6	3.1	(1.1–6.8)	3	1.7	(0.3–4.9)	3	1.5	(0.3–4.3)	7	2.9	(1.2–6.0)	3	2.7	(0.6–7.9)
*Chronic myeloid leukemia*	0	0.0		1	1.2	(0.0–6.8)	0			0	0.0		0	0.0		0	0.0	
Unknown primary	6	1.7	(0.6–3.7)	4	0.6	(0.2–1.6)	6	0.8	(0.3–1.8)	3	1.5	(0.3–4.3)	0	0.0		7	1.9	(0.8–3.9)
Total combined^a^	245	1.1	(1.0–1.2)	305	0.9	(0.8–1.0)	202	0.7	(0.6–0.8)	87	1.2	(1.0–1.5)	124	1.4	(1.2–1.7)	138	1.2	(1.0–1.5)

Only cancers shown in Table 2 are presented.

^*^Rounding off for SIRs above 10; Bold type (elevated risk) and underscored type (decreased risk): 95% CIs did not include 1.00.

^a^Including cancers not presented individually.

**Table 4:** SIRs of second primary cancers in survivors of multiple myeloma (MM) by follow–up time after first MM in Germany and Sweden*.

Sites of second cancers	Germany									Sweden								
	<1 yr			1–4 yrs			≥5 yrs			<1 yr			1–4 yrs			≥5 yrs		
	N	SIR	95%CI	N	SIR	95%CI	N	SIR	95%CI	N	SIR	95%CI	N	SIR	95%CI	N	SIR	95%CI
Stomach	9	0.9	(0.4–1.6)	21	1.0	(0.6–1.6)	11	1.7	(0.9–3.1)	1	0.5	(0.0–2.8)	7	1.9	(0.8–3.9)	0		
Colorectum	18	0.5	(0.3–0.7)	60	0.8	(0.6–1.0)	26	1.1	(0.7–1.6)	13	1.0	(0.5–1.8)	38	1.6	(1.1–2.1)	6	0.9	(0.3–1.9)
Liver and gallbladder	5	0.8	(0.3–1.8)	14	1.1	(0.6–1.8)	4	0.9	(0.3–2.4)	2	1.8	(0.2–6.5)	3	1.6	(0.3–4.6)	1	0.9	(0.0–5.2)
Lung	24	0.8	(0.5–1.3)	41	0.7	(0.5–1.0)	19	1.1	(0.6–1.7)	7	1.6	(0.6–3.3)	9	1.1	(0.5–2.1)	6	1.3	(0.5–2.9)
Breast	7	0.3	(0.1–0.6)	49	1.0	(0.7–1.3)	13	0.8	(0.4–1.3)	5	0.8	(0.2–1.8)	19	1.2	(0.7–1.9)	6	1.3	(0.5–2.8)
Endometrium	5	0.9	(0.3–2.2)	3	0.3	(0.1–0.8)	0			0			3	1.4	(0.3–4.1)	2	1.2	(0.1–4.2)
Prostate	37	0.9	(0.6–1.2)	55	0.6	(0.5–0.8)	25	0.9	(0.6–1.3)	22	1.2	(0.8–1.8)	28	1.1	(0.7–1.6)	15	1.6	(0.9–2.6)
Kidney	10	1.1	(0.5–2.1)	18	1.0	(0.6–1.6)	7	1.2	(0.5–2.5)	7	3.1	(1.2–6.4)	10	1.9	(0.9–3.6)	0		
Urinary bladder	5	0.4	(0.1–1.0)	13	0.6	(0.3–1.0)	5	0.7	(0.2–1.6)	5	1.0	(0.3–2.2)	8	0.8	(0.4–1.6)	6	1.4	(0.5–3.0)
Melanoma	6	1.0	(0.3–2.1)	18	1.4	(0.8–2.2)	7	1.7	(0.7–3.4)	1	0.3	(0.0–1.4)	16	1.9	(1.1–3.2)	5	1.5	(0.5–3.5)
Nervous system	3	1.2	(0.2–3.5)	7	1.4	(0.6–2.8)	2	1.2	(0.1–4.2)	6	3.0	(1.1–6.6)	8	1.4	(0.6–2.7)	3	3.2	(0.7–9.5)
Non–Hodgkin lymphoma	11	1.5	(0.8–2.8)	8	0.6	(0.2–1.1)	3	0.7	(0.1–1.9)	1	0.4	(0.0–2.0)	13	2.2	(1.2–3.7)	2	1.3	(0.2–4.5)
Leukemia	8	1.4	(0.6–2.8)	18	1.6	(1.0–2.6)	9	2.5	(1.2–4.8)	7	1.7	(0.7–3.5)	13	1.8	(1.0–3.1)	4	1.1	(0.3–2.9)
Myeloid leukemia	6	2.7	(1.0–5.8)	16	3.7	(2.1–5.9)	9	6.3	(2.9–12)	2	1.4	(0.2–5.0)	9	2.3	(1.0–4.3)	3	1.8	(0.4–5.2)
*Acute myeloid leukemia*	4	3.0	(0.8–7.7)	13	5.0	(2.7–8.6)	7	8.2	(3.3–17)	2	1.9	(0.2–6.8)	8	2.7	(1.2–5.4)	3	2.0	(0.4–5.8)
*Chronic myeloid leukemia*	1	1.7	(0.0–9.3)	0	0.0		0	0.0		0	0.0		0	0.0		0		
Unknown primary	5	1.0	(0.3–2.4)	9	0.9	(0.4–1.8)	2	0.6	(0.1–2.3)	2	0.9	(0.1–3.1)	7	1.7	(0.7–3.6)	1	6.1	(0.2–34)
Total combined^a^	198	0.8	(0.7–1.0)	402	0.9	(0.8–1.0)	152	1.0	(0.8–1.2)	88	1.1	(0.9–1.4)	199	1.4	(1.2–1.6)	62	1.3	(1.0–1.7)

Only cancers shown in Table 2 are presented.

^*^Rounding off for SIRs above 10; Bold type (elevated risk) and underscored type (decreased risk): 95% CIs did not include 1.00.

^a^Including cancers not presented individually.
